# Level of knowledge and practice of female healthcare providers about early detection methods of breast cancer at Debre Tabor Comprehensive Specialised Hospital: a cross-sectional study

**DOI:** 10.3332/ecancer.2021.1268

**Published:** 2021-07-19

**Authors:** Aragaw Tesfaw, Hanna Berihun, Eshetie Molla, Gashaw Mihret, Dejen Getaneh Feleke, Ermias Sisay Chanie, Biruk Demissie, Tewodros Yosef, Abel Shita, Fitalew Tadele, Efrem Fenta

**Affiliations:** 1 Department of Public Health, College of Health Sciences, Debre Tabor University, Debre Tabor, Ethiopia; 2 College of Health Sciences, School of Medicine, Debre Tabor University, Debre Tabor, Ethiopia; 3 Department of Pediatrics and Child Health Nursing, College of Health Sciences, Debre Tabor University, Debre Tabor, Ethiopia; 4 Department of Epidemiology and Biostatistics, College of Medicine and Health Sciences, Mizan Tepi University, Mizan Teferi, Ethiopia; 5 Department of Public Health, Mizan Aman College of Health Sciences, Mizan Aman, Ethiopia; 6 Department of Biomedical Sciences, College of Health Sciences, Debre Tabor University, Debre Tabor, Ethiopia; 7 Department of Anaesthesia, College of Health Sciences, Debre Tabor University, Debre Tabor, Ethiopia

**Keywords:** knowledge, practice, breast cancer, healthcare providers, Ethiopia

## Abstract

**Background:**

Despite the higher mortality rate of breast cancer in low and middle-income countries, the practice of early detection methods is low and the majority of the patients presenting at an advanced stage of the disease need palliative care with low survival rates. Although healthcare providers are the key for practicing early detection methods of breast cancer for themselves and their clients, little is known about their knowledge and practice of early detection methods of breast cancer in Northcentral Ethiopia.

**Methods:**

An institution-based cross-sectional study was conducted among female healthcare providers at Debre Tabor Comprehensive Specialised Hospital. Data were collected using a structured self-administered questionnaire. The data were analysed using SPSS version 23. Descriptive statistics were used to describe the socio-demographic information of participants. Binary and multivariable logistic regression with adjusted odds ratio (AOR) and 95% confidence interval (CI) was used to identify factors associated with the outcome variable. Statistical significance was declared at *p* < 0.05.

**Result:**

The mean (±SD) age of the participants was 31.4 (±7.8) years. About 106 (79.7%) participants had good knowledge about breast cancer early detection methods and only 56 (42.1%) of them practiced breast self-examination regularly. Work experience of >2 years (AOR = 3.2; 95% CI: 1.72, 5.29), history of any breast problem (AOR = 1.4; 95% CI: 1.02, 2.37), family history of breast cancer (AOR = 4.0; 95% CI: 2.58, 15.84), having good knowledge (AOR = 2.9; 95% CI: 1.3, 6.52) and history of comorbidities (AOR = 1.09; 95% CI: 1.09, 3.59) were the factors associated with the practice of breast self-examination.

**Conclusion:**

Our study found that the knowledge and practice of breast cancer early detection methods was low in the study setting. Only less than half of female healthcare providers practiced regular breast self-examination, which suggests the need to provide training for healthcare providers to fill the gap and to promote early detection of breast cancer cases.

## Background

Breast cancer is the most common malignant tumour which can be characterised by distinct clinical, pathologic and molecular characteristics [1, 2]. It is a growing public health concern globally as the leading cause of cancer with high mortality rates in low and middle-income countries [3]. Previously, breast cancer mainly occurred in developed countries, but nowadays the incidence has changed and it has become a public health threat to developing countries also. Almost 50% of breast cancer cases and 58% of deaths occur in low and middle-income countries (LMICs) [4]. Female breast cancer has now surpassed lung cancer and has become the leading cause of global cancer incidence in 2020, with an estimated 2.3 million new cases. The incidence of new breast cancer cases in Ethiopia is now increasing. It is the commonest cancer constituting 33% of the cancers in women and 23% of all cancers in the county [5, 6].

Even though early diagnosis of cancer with accessible, affordable and effective treatment results in improvements in both the stage of cancer at presentation and mortality from cancer, many healthcare systems in LMICs are least prepared to manage the burden of cancer and patients do not have access to timely, high-quality diagnosis or treatment. In addition, little community awareness, inadequate advanced pathology services and fragmented treatment options are the biggest challenges for low-income countries [2]. The principles to achieve early diagnosis include increasing cancer awareness and health participation, promoting accurate clinical evaluation, pathologic diagnosis, staging and improving access to care [7].

Early detection and treatment are critical for reducing mortality rates and increasing survival of breast cancer patients. Currently, there are three screening methods recommended for early detection of breast cancer: clinical breast examination (CBE), mammography and breast self-examination (BSE) [3]. BSE is a simple, non-invasive and inexpensive method of early detection which is recommended for all women who are 20 years of age to identify any abnormalities or changes in their breast. It is mainly important for women in developing countries where access to health professionals and oncology services is limited [3, 7].

Healthcare provider knowledge about early detection methods is crucial for preventing delayed diagnosis and advanced stage presentation due to inadequate patient examination, use of inappropriate tests or missed interpretation of test results, misdiagnosis either through treating patients symptomatically or relating symptoms to a health problem other than cancer [8]. A similar study carried out among female healthcare professionals in Ethiopia found poor practice of BSE (35.5%), CBE (32.5%) and mammography (16%) among the providers [9].

In Ethiopia, there is a plan to control the increasing trends of breast cancer through screening and early detection programs such as mammography, clinical and breast self-examination, but there is also a need to build breast cancer diagnostic centres in different parts of the country to know the number of cases, which are being misdiagnosed due to lack of accessibility and availability of facilities [10, 11].

In 2018, over 15,000 women developed breast cancer in Ethiopia and around 8,000 died in the same year. Breast cancer is three times deadly for women in Ethiopia than in the United States [12]. The most common clinical presentation of breast cancer is a hard lump. Advanced diseases may have skin in drawing with colour change and metastatic symptoms [13]. Triple assessment, which includes clinical examination, imaging and a pathology study, is the best way to detect any suspected carcinoma of the breast with a 99% predictive rate [1].

Early diagnosis is the main way of preventing cancer death in low-income countries as prevention of risk factors and treatment is difficult. Breast self-examination is among one of the early diagnostic ways of breast cancer helping to raise awareness of the disease [14].

The main barriers causing the disparity in cancer outcome in Ethiopia are the shortage of trained workers and long distance between treatment facilities [11, 15]. Currently, there are only 13 oncologists and 18 pathologists for the whole population of 100 million with only a few cancer centres showing the gap in cancer care. The Ethiopian government has been working to maximise the level of care by constructing six new regional oncology centres with advanced ways of treatment. In addition to this, the country has long-term plans to train more oncologist specialist, nurses and pharmacists [12].

In a study conducted among nurses in Addis Ababa University, Ethiopia, while asked about screening methods, around two-thirds of them mentioned BSE, while others listed CBE and mammography. The study also concluded that majority of them knew about the importance of early detection on the prognosis and that breast cancer can be prevented at early stage of the disease [16]. A cross-sectional study on health extension workers in Addis Ababa found that more than half of the participants had adequate knowledge of BSE. Breast lump, nipple discharge, pain and breast size change were the main symptoms mentioned [17].

With increasing prevalence in developing countries, breast cancer is becoming a major public health problem in Ethiopia [18]. A reviewed literature showed that there is a knowledge and screening practice gap among women healthcare providers in developing countries including Ethiopia with minimal available healthcare institutions to help affected patients. In addition, some studies reported from different corners of the country showed that there is a variation in the knowledge and practice of early detection methods among healthcare providers. However, evidence-based information about the level of knowledge and practice of breast cancer early detection methods among healthcare providers is lacking in our study area. Therefore, our study aimed to assess the knowledge and practice of female healthcare providers on breast cancer early detection method at Debre Tabor Comprehensive Specialised Hospital, Northcentral, Ethiopia.

## Methods

### Study design and area

The study was conducted from November 10–30, 2020, at Debre Tabor Comprehensive Specialised Hospital, the largest hospital in the zone, which has been providing services to 2.3 million people in its catchment area. More than 30 medical specialists in various areas of specialisation and a fairly adequate number of all other healthcare professionals constitute the healthcare team. The hospital is now emerging as a shining hot spot for advanced medical care and treatment in the northcentral parts of Ethiopia with a total capacity of 110 inpatient beds in five major departments and a total of more than 101,357 patient flows per year. In addition, the hospital is used as a teaching hospital for medical and health science students of Debre Tabor University. The hospital has a moderate infrastructure to diagnose and treat breast cancer patients. It has ultrasonography and fine needle aspiration cytology investigations and surgical teams to treat the disease. The hospital refers patients for chemotherapy and radiotherapy services to Felege Hiwot Referral Hospital and Tikur Anbesa Specialised Hospitals, respectively.

### Study population and data collection procedures

The study population were all women healthcare providers currently working at Debre Tabor General Hospital. The data were collected using self-administered structured questionnaires which were developed from the reviewed literature [15, 19, 20]. The questionnaires consisted of socio-demographic, medical and reproductive history and knowledge and practice assessment questions. Two supervisors were assigned for supervising the data collection process in the hospital.

### Sample size determination and sampling technique

The sample size was determined using a single population proportion formula by taking the margin of error 5%, confidence level 95% and proportion of practicing breast self-examination (66.8%) from a study carried out among healthcare workers in the West Shoa zone [15] as follows:

*N* = *Z*^2^ × *P* × (1−*p*)/*d*^2^

= 1.96^2^ × 0.668 × 0.332/0.05^2^

=133

Those 133 women were selected by systematic random sampling from a total of 216 women healthcare providers. This was carried out by dividing the total number of participants by the sample size which was 133: 216/133 = 2, and then the sample selected every other woman from the total women staff in the hospital.

### Operational definitions

**Early detection methods of breast cancer:** Breast self-examination, clinical breast examination and mammography are the early detection methods of breast cancer [7, 21].

**Practice of breast self-examination:** Those who carried out breast self-examination practice a week after each menses used their palm and middle three fingers [22].

**Clinical breast examination:** This was measured to see whether participants had at least one clinical check-up in the last 1-year period.

**Mammography check-ups:** This was measured to see whether the participants had at least one mammography check-up in their life time.

**Good knowledge about breast cancer early detection methods:** Participants were considered as having good knowledge if they answered more than the mean score of the knowledge assessment questions.

**Poor knowledge about breast cancer early detection methods:** Participants were considered as having poor knowledge if they answered less than the mean score of the knowledge assessment questions.

### Data quality and analysis procedures

The quality of the data was maintained by using a structured and pretested questionnaire. The questionnaire was prepared in a simple and easily understandable language which was initially prepared in English language and later translated to the local language (Amharic) to facilitate communication. A pre-test was conducted prior to actual data collection outside the study hospital. After pretesting, the difficult questions were revised and modification was carried out according to the expert’s judgment on the clarity of sentences and appropriateness of content. After adjustment of the questions, the actual data collection was conducted by using the Amharic version questionnaire. The pretested data were not included in the main data.

Before analysis, the data were checked for completeness and internal consistency, then it was coded and entered using Epi Info version 7.2 and analysed using SPSS version 23. Descriptive statistical analysis was used to present the socio-demographic and clinical characteristics of the study participants. Binary logistic regression was used to measure the association of each factor with breast self-examination. In addition, factors that were associated with the outcome variable at the 25% significance level were included in the multivariable logistic regression analysis to control the potential confounders. An adjusted odds ratio (AOR) and 95% confidence interval (CI) were used to describe the final model. *P*-value less than 0.05 was used to identify statistically significant results.

### Ethical consideration

An ethical approval letter was obtained from a research ethics committee of the College of Health Science, Debre Tabor University. The medical director of the hospital was informed through a support letter. Oral informed consent was obtained from the participants after a detailed explanation was given about the objectives and benefits of the study. The confidentiality of information was respected.

## Result

### Socio-demographic characteristics of study participants

A total of 133 healthcare providers from different health science disciplines participated in the study. The mean age of the participants was 31.4 ± 7.8 years. Nearly half (66; 49.6%) of the participants were in the age group of 30–39 years. A higher proportion (86; 64.7%) of healthcare providers was married. Majority (64; 48.1%) of healthcare providers were nurses. More than half (78; 58.6%) of the providers had more than 2 years of work experience in the hospital (Table 1)**.**

### Medical and reproductive characteristics of study participants

About 14 (10.5%) healthcare providers had a family history of breast cancer and 29 (21.8%) of them had a personal history of some breast disease. More than one-fourth (44; 33.1%) of the participants have a history of comorbidities (diabetes mellitus, hypertension and tuberculosis). About 68 (51.1%) providers had a history of giving birth (Table 2).

### Knowledge and practice of female healthcare providers about early detection methods of breast cancer

The majority (132, 99.2%) of female healthcare providers have ever heard about breast self-examination. About 112 (84.2%) agreed on the importance of breast self-examination for early detection of breast cancer. Regarding the knowledge assessment, about 106 (79.7 %) participants had good knowledge about early detection methods of breast cancer. More than half (76; 57.1%) of the healthcare providers said BSE should be appropriate at the age of 41–60 years. About 59 (44.1%) of the participants have agreed that BSE should be carried out every month after menses. More than three-fourth (116; 87.2%) of the healthcare providers stated that BSE should be carried out by using the palm and three finger palpation, while 15 (11.3%) of them did not know how BSE is carried out (Table 3).

**Figure 1.**
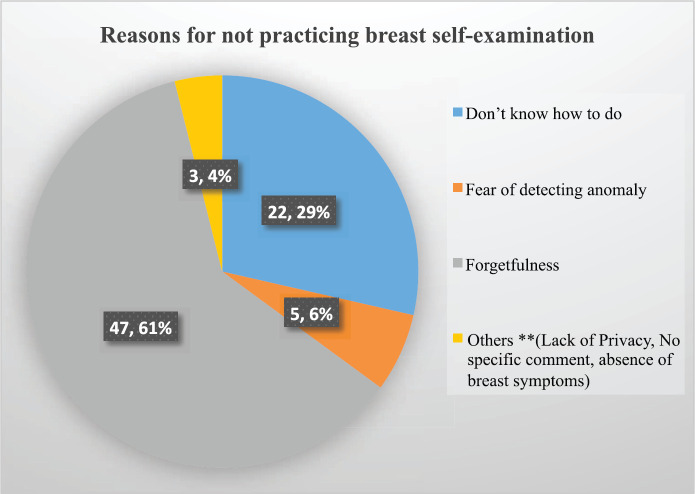
Reasons for not practicing breast self-examination among female healthcare providers in Northcentral Ethiopia, 2020.

About 37 (27.8%) healthcare providers had a history of clinical breast examination, while only 8 (6%) participants had a history of mammography check-ups and about 112 (84.2%) claim that they have carried out BSE in the past 12 months, while only 56 (42.1%) healthcare providers regularly practiced breast self-examination every month. The major reason for not practicing breast self-examination was forgetfulness (47; 61%), followed by ‘do not know how to do breast self-examination’ (22; 29%) (Figure 1).

### Factors associated with the practice of breast self-examination

In a multivariable logistic regression analysis, healthcare providers who have more than 2 years of work experience, good knowledge about breast cancer, early detection methods, family history of breast cancer, history of any breast disease and a history of comorbidities were significantly associated with the practice of breast self-examination. Participants who have more than 2 years of work experience were 3.2 times more likely to practice breast self-examination than those who have less than 2 years of work experience (AOR = 3.2; 95% CI: 1.72, 5.29). Participants who have good knowledge about breast cancer early detection methods were 2.9 times more likely to practice breast self-examination than those who have a poor knowledge (AOR = 2.9; 95% CI: 1.3, 6.52). Healthcare providers who have a family history of breast cancer were four times more likely to practice breast self-examination than those who do not have a family history of breast cancer (AOR = 4.0; 95% CI: 2.58, 15.84). Healthcare providers who have a history of any breast disease were 1.4 times more likely to practice breast self-examination than who did not have a history of any breast disease before (AOR = 1.4; 95% CI: 1.02, 2.37). Healthcare providers who have a history of any comorbidities were 1.9 times more likely to practice breast self-examination than who did not have a history of any comorbidities (AOR = 1.09; 95% CI: 1.09, 3.59) (Table 4).

## Discussion

Most breast cancer patients in low and middle-income countries presented after the tumour was metastasised to other body parts which significantly affects their survival. Most breast cancer patients in developing countries suffer very long delays and have high mortality rates [23–26]. Although healthcare providers are the key for creating awareness about breast cancer and its early detection methods of breast cancer, most studies reported as that there is a gap in the knowledge and practice of those early detection methods [27–29].

Our study revealed that the practice of breast cancer early detection methods is low among female healthcare providers in the study setting, although majority of them had good knowledge about the methods. Most of the participants mentioned the early detection methods of breast cancer as mammography (28.6%), clinical breast self-examination (95.5%) and breast self-examination (99.2%), which is higher than the study mentioned in West Shoa [17]. The discrepancy might be due to variation in study population characteristics. In addition, the study period difference might be linked with increased awareness.

Our study found that the practice of regular (every month) breast self-examination among healthcare providers was 42.1%, which is nearly in line with a study conducted in a tertiary educational institution at Addis Ababa [30]. However, it was lower than the study conducted among Bahirdar University’s female students (54.1%) [31] and higher than a study conducted among healthcare providers in West Shoa (32.6%) [17]. This study also revealed that about 106 (79.7%) healthcare providers had good knowledge about early detection methods of breast cancer. This finding is consistent with the study on Bahirdar University’s female students [31] but lower than a study from West Shoa (66.8%) [17], Eritrea (60.6%) [32] and the pooled prevalence estimate in Ethiopia [28]. The difference might be due to the difference in the study participants and study period.

In this study, participants who have more than 2 years of work experience were 3.2 times more likely to practice breast self-examination than who have less than 2 years of work experience. However, there was no significant association found between work experience and BSE practice in a study carried out in West Shoa [17]. The variation might be due to the difference in sample size and work experience between the studies. Similarly, participants who have good knowledge about breast cancer early detection methods were 2.9 times more likely to practice breast self-examination than those who have a poor knowledge. This finding is similar to a study conducted in West Shoa Debre Birhan University and Turkey [17, 33, 34]. This might be explained by the fact that having knowledge could increase individuals’ self-confidence and help them gain experience; and this will initiate women to practice breast self-examination.

In our study, healthcare providers who have a family history of breast cancer were four times more likely to practice breast self-examination than who do not have a family history of the problem. This finding is in line with a study conducted in Addis Ababa and a meta-analysis finding in Ethiopia [28, 35]. This might be because the providers may perceive that they are at risk of having the disease in their lifetime once they have a family history of the disease.

This study also revealed that healthcare providers who had a history of any breast disease were 1.4 times more likely to practice breast self-examination than who did not have a history of any breast disease. History of any comorbidity was also significantly associated with the practice of breast self-examination. Healthcare providers who had a history of comorbidities were more likely to practice breast self-examination than who did not have a history of comorbidities. This might be due to the fear that having comorbidities and any breast problems may induce participants to check their breasts. In addition, the positive association between family history of breast cancer, comorbidities and history of breast abnormalities could be due to the realisation of female healthcare providers to develop the consequences of the disease. Moreover, they might see themselves under risk for the disease and believe in the importance of screening for early stage diagnosis. The finding is in line with other study findings [28].

### Strengths and limitations of the study

Based on the knowledge of the researchers, this study is the first study to be conducted in the area which showed the level of knowledge and practice of breast cancer early detection methods among female healthcare providers. Even though we failed to account for non-respondents in our sample calculation, which is an inherent limitation of data collected using self-administered questionnaires, our response rate was 100%. Moreover, our *post-hoc* power calculation was >80%. However, our study might be affected by recall bias since participants face some difficulties to remember their practice on breast self-examination. It is also difficult to establish a temporal relationship between the factors and outcome due to the cross-sectional nature of the study.

## Conclusion

In conclusion, the knowledge and practice of breast cancer early detection methods was low in the study area. Only less than half of the female healthcare providers practice regular breast self-examination. More than 2 years of work experience, having good knowledge about breast cancer, early detection methods, family history of breast cancer, history of any breast disease and a history of comorbidities were the factors significantly associated with the practice of breast self-examination. The findings of this study suggest the need to provide short-term training for healthcare providers to fill in the knowledge gaps on the practice of breast cancer early detection methods since they should be role models for the rest of the community to promote early diagnosis and downstage breast cancer.

## Ethics approval and consent to participate

An ethical approval letter was obtained from a research ethics committee of the College of Health Science, Debre Tabor University. The medical director of the hospital was informed through a support letter. Oral informed consent was obtained from participants after a detailed explanation was given about the objectives and benefits of the study. The confidentiality of information was respected.

## Consent for publication

Not applicable.

## Availability of data and materials

The necessary data are available in the main manuscript document and its supporting information file.

## Competing interests

The authors declare that they have no competing interests.

## Funding

Not applicable.

## Authors’ contributions

AT and HB were involved in the initiation of the idea, write-up of the proposal, data collection, data entry, data analysis and final manuscript write-up, while EM, GM, DG, EC, EF, FT, BD, AS and TY were involved in supervision, manuscript editing and write-up. All authors were involved in the revision of the manuscript and approval of the final revised manuscript.

## Figures and Tables

**Table 1.** Socio-demographic characteristics of female healthcare providers at Debre Tabor Comprehensive Specialised Hospital, Northcentral Ethiopia, 2020.

Characteristics	Frequency	Percentage
**Age group**		
<30	37	27.8
30-39	66	49.6
40-49	18	13.5
≥50	12	9.0
Mean ± SD	31.4 ± 7.8 years	
**Religion**		
Orthodox	106	79.7
Muslim	13	9.8
Protestant	8	6.0
Catholic	6	4.5
**Marital status**		
Married	86	64.7
Single	47	35.3
**Level of education**		
Diploma	45	33.8
Degree	67	50.4
Masters	21	15.8
**Type of profession**		
Nurses	64	48.1
Medical doctors	28	21.1
Others^a^	41	30.8
**Work experience**		
<2 years	55	41.4
≥2 years	78	58.6

a Medical laboratory technician, pharmacist, anaesthetist and midwifery

**Table 2.** Reproductive and medical history of female healthcare providers at Debre Tabor Comprehensive Specialised Hospital, Northcentral Ethiopia, 2020.

Characteristics	Frequency	Percentage
**Pregnancy status**		
Not pregnant	122	91.7
Pregnant	11	8.3
**Family history of breast cancer**		
Yes	14	10.5
No	119	89.5
**History of any breast disease**		
Yes	29	21.8
No	104	78.2
**History of any comorbidities**		
Yes	44	33.1
No	89	66.9
**History of giving birth**		
Yes	68	51.1
No	65	48.9
**History of contraceptives**		
Yes	97	72.9
No	36	27.1
**History of alcohol**		
Yes	26	19.5
No	107	80.5

**Table 3.** Knowledge and practice of female healthcare providers about early detection methods of breast cancer at Debre Tabor Comprehensive Specialised Hospital, Northcentral Ethiopia, 2020.

Characteristics	Category	Frequency	Percentage
Which breast cancer early detection method have you ever heard of?	Breast self-examination	132	99.2
	Clinical breast examination	127	95.5
	Mammogram	38	28.6
Do you think BSE is important for early detection of breast cancer?	Yes	112	84.2
	No	21	15.8
Which is the appropriate time of BSE for early detection of breast cancer	Few days before menses	74	55.6
	Few days after menses	102	76.7
	No specific time	6	4.5
	Other^a^ (I do not know, during menses)	13	9.8
Knowledge about breast cancer early detection methods	Good knowledge	106	79.7
	Poor knowledge	27	20.7
Perceived risk factors for breast cancer	Family history of breast cancer	127	95.5
	Early menarche	105	78.9
	Radiation/hazardous chemical exposure	98	7.4
	Advanced age	113	84.9
	Overweight after menopause	65	48.9
	Prolonged use of oral contraceptives	79	59.4
	Others^b^	24	18.0
How is BSE is carried out	One finger palpation	2	1.5
	Palm and three finger palpation	116	87.2
	Don’t know	15	11.3
At what age BSE should be carried out	<20 years	4	3.0
	20–40 years	22	16.5
	41–60 years	76	57.1
	>60 years	28	21.1
	Don’t know	3	2.3
Frequency of BSE	Monthly	59	44.4
	Every 3 months	34	25.6
	Every 6 months	25	18.8
	Once a year	6	4.5
	Don’t know	9	6.8
Knowledge about breast self-examination	BSE is the assessment made on the breast by an individual to check for breast lump	132	99.2
	BSE should be carried out every month	59	44.4
	Examining breasts is carried out 1–7 days after the end of menstrual period	102	76.7
	BSE is carried out looking at breasts in the mirror	45	33.8
	BSE is carried out with arms raised over head	38	28.6
	Examining one’s breast is possible while lying down	12	9.0
	In BSE, the women need to look for lumps using tips of fingers	87	65.4
	BSE is carried out in a circular, clockwise motion moving from outside in	78	58.6
	In BSE, the women need to squeeze the nipples of each breast to look for discharge	54	40.6
	When examining breast, feel for lumps, thickening and lumps under armpits	128	96.2
	Examining breast should begin at age 20	36	27.1

Other^a^ =I do not know, during menses; Others^b^ = smoking, alcohol, pregnancy after 30 years and family history of breast cancer

**Table 4.** Bivariate and multivariable logistic regression analyses for factors associated with the practice of breast self-examination among healthcare providers in Northcentral Ethiopia, 2020.

Determinant factors	Practice of BSE		COR with 95% CI	AOR with 95% CI	*p*-value
	Yes	No			
**Age group**					
<30	14 (37.8%)	23 (62.2%)	1	1	
30–39	22 (33.3)	44 (66.7)	0.82 (0.35, 1.90)	1.4 (0.64, 3.03)	0.404
40–49	12 (66.7%)	6 (33.3%)	3.3 (1.01, 10.74)	1.3 (0.11, 8.35)	0.264
≥50	8 (66.7%)	4 (33.3%)	3.2 (0.83, 12.93)	0.9 (0.37, 2.43)	0.924
**Level of education**					
Diploma	18 (40%)	27 (60%)	1	1	
Degree	24 (35.8%)	43 (64.2%)	0.83 (0.38, 1.82)	1.6 (0.73, 3.87)	0.221
Masters	14 (66.7%)	7 (33.3%)	3.0 (1.01, 8.88)	0.72 (0.02, 2.96)	0.621
**Work experience**					
<2 years	29 (52.7%)	26 (47.3%)	1	1	**<0.001**
≥ 2 years	27 (34.6%)	51 (65.4%)	2.1 (1.04, 4.26)	3.2 (1.72, 5.29)	
**Knowledge about early detection methods**					
Good knowledge	36 (33.9%)	70 (66.1%)	5.5 (2.2, 14.34)	2.9 (1.30, 6.52)	**0.009**
Poor knowledge	20 (74.1%)	7 (25.9%)	1	1	
**Family history of breast cancer**					
Yes	12 (85.7%)	2 (14.3%)	10.2 (2.2, 47.83)	4.0 (2.58, 15.84)	**0.001**
No	44 (36.9%)	75 (63.1%)	1	1	
**History of any breast problem before**					
Yes	18 (62.1%)	11 (37.9%)	2.8 (1.22, 6.65)	1.4 (1.02, 2.37)	**0.021**
No	38 (36.5%)	66 (63.5%)	1	1	
**Any comorbidities**					
Yes	21 (47.7%)	23 (52.3%)	9.8 (3.31, 29.34)	1.9 (1.09, 3.59)	**0.024**
No	5 (39.3%)	54 (60.7%)	1	1	


COR=Crude odds ratio; AOR=Adjusted Odds Ratio

Bold indicates statistical significant association
